# RP-Rs-fMRIomics as a Novel Imaging Analysis Strategy to Empower Diagnosis of Brain Gliomas

**DOI:** 10.3390/cancers14122818

**Published:** 2022-06-07

**Authors:** Xiaoxue Liu, Jianrui Li, Qiang Xu, Qirui Zhang, Xian Zhou, Hao Pan, Nan Wu, Guangming Lu, Zhiqiang Zhang

**Affiliations:** 1 Department of Diagnostic Radiology, Affiliated Jinling Hospital, Medical School of Nanjing University, Nanjing 210002, China; fmriliuxx@163.com (X.L.); ljr1984618@163.com (J.L.); fmrixuq@126.com (Q.X.); fmrizhangqr@126.com (Q.Z.); yaoyaomao13@163.com (X.Z.); cjr.luguangming@vip.163.com (G.L.); 2 Department of Neurosurgery, Affiliated Jinling Hospital, Medical School of Nanjing University, Nanjing 210002, China; panhao_nz@163.com; 3 Department of Pathology, Affiliated Jinling Hospital, Medical School of Nanjing University, Nanjing 210002, China; 55152572@163.com; 4 State Key Laboratory of Analytical Chemistry for Life Science, Nanjing University, Nanjing 210093, China

**Keywords:** resting-state fMRI, regional parameter, glioma, RP-Rs-fMRIomics

## Abstract

**Simple Summary:**

Resting-state functional magnetic resonance imaging (rs-fMRI), a popular neuroimaging technique, can provide rich information about functional processes in the brain with a large array of imaging parameters and is suitable for exploring the pathophysiological essence of gliomas. In this study, by applying omics analysis strategy to rs-fMRI with exhaustive regional parameters, we proposed a novel approach, named Regional Parameter of Resting-state fMRI-omics (RP-Rs-fMRIomics), and further evaluated the diagnosis performance of the method on brain gliomas. We found that the RP-Rs-fMRIomics, featuring entire investigation and high interpretability, presented superior performance in prediction of tumor grade, IDH genotype and prognosis of brain gliomas. This RP-Rs-fMRIomics not only contributed a new imaging method for brain glioma research, but also expanded the clinical application of rs-fMRI.

**Abstract:**

Rs-fMRI can provide rich information about functional processes in the brain with a large array of imaging parameters and is also suitable for investigating the biological processes in cerebral gliomas. We aimed to propose an imaging analysis method of RP-Rs-fMRIomics by adopting omics analysis on rs-fMRI with exhaustive regional parameters and subsequently estimating its feasibility on the prediction diagnosis of gliomas. In this retrospective study, preoperative rs-fMRI data were acquired from patients confirmed with diffuse gliomas (*n* = 176). A total of 420 features were extracted through measuring 14 regional parameters of rs-fMRI as much as available currently in 10 specific narrow frequency bins and three parts of gliomas. With a randomly split training and testing dataset (ratio 7:3), four classifiers were implemented to construct and optimize RP-Rs-fMRIomics models for predicting glioma grade, IDH status and Karnofsky Performance Status scores. The RP-Rs-fMRIomics models (AUROC 0.988, 0.905, 0.801) were superior to the corresponding traditional single rs-fMRI index (AUROC 0.803, 0.731, 0.632) in predicting glioma grade, IDH and survival. The RP-Rs-fMRIomics analysis, featuring high interpretability, was competitive for prediction of glioma grading, IDH genotype and prognosis. The method expanded the clinical application of rs-fMRI and also contributed a new imaging analysis for brain tumor research.

## 1. Introduction

Resting-state functional magnetic resonance imaging (rs-fMRI) has been increasingly the most popular neuroimaging technique for investigating human brain function in physiological and pathological states [1]. By measuring low-frequency blood oxygen level dependent (BOLD) fluctuations with a growing array of parameters, rs-fMRI provides rich information for depicting features of spontaneous brain activity from connectivity and regional aspects [2,3,4]. Connectivity measures interregional relationships and is dedicated to the construction of functional brain networks [5,6]. On the contrary, regional parameters, including amplitude of low-frequency fluctuation (ALFF) [7], regional homogeneity (ReHo) [8,9], Hurst exponent (HE) [10] and time-shift-analysis (TSA) [11], voxel-wisely describe the local brain activity in amplitude, frequency and temporal profiles.

Rs-fMRI has also been extensively applied to brain gliomas in clinics. The values of rs-fMRI have been well established in mapping eloquent regions and estimating cognitive function for presurgical planning [12,13]. Moreover, recent investigation has increasingly expanded rs-fMRI to the pathophysiological essence of the tumors by taking advantage of the features of rich information and versatile measures in the technique. In particular, studies have applied rs-fMRI to classify gliomas through observing network characteristics [5,12,14]. However, the network metrics, which measure signals mostly outside the tumor at the whole brain level, might not be specific to the nature of gliomas under a black-box model. A few of other studies observed regional parameters of rs-fMRI within the tumor itself [14,15,16]. Metwali et al. [14] adopted ALFF to investigate tumor grades and found that high-grade gliomas showed significantly higher amplitudes of fluctuation compared with low-grade gliomas. Gupta et al. [16] combined the temporal shift, ALFF and ReHo to characterize the BOLD signal in gliomas and found that the temporal shift and ALFF were significantly distinguishable between high- and low-grade gliomas. These phenotypes of regional parameters within the tumor have been illustrated as intratumoral changes in oxygenation and hemodynamics caused by vascular dysregulation and venous effects in gliomas [14,16,17,18,19,20,21], suggesting that BOLD signals could reflect the biological characteristics of gliomas.

However, previous studies have mostly adopted univariate analysis on one or several imaging parameters separately, and the adopted parameters were all based on conventional frequency bands (approximately 0.01–0.08 Hz) of BOLD signals. The operations would fail to take full advantage of the fMRI nature with versatile measures and rich intrinsic information [22]. Accordingly, a concept of biomedicine analysis strategy, omics, can comprehensively measure the profiles of structure and functions of the whole makeup of a given biological function by analyzing large amounts of data representing an entire set of genes, proteins, metabolism, etc. [23]. Notably, a recent omics analysis method in medical imaging, radiomics [24,25], can introduce thousands of high-throughput features at one time, followed by various methods for feature selection and model building to seek the best combination of features for clinical prediction, which has the advantage of making full use of multiple indicators. However, feature extraction in radiomics is based on measuring the intensity and morphological characteristics, and the data-driven nature of radiomics inherently offers no insight into the biological underpinnings of observed relationships [26]. A few recent fMRI studies [27,28,29,30] innovatively borrowed tactics of radiomics analysis. Features were extracted from parameters within multiple brain parcellations or based on constructed connectivity between the brain parcellations. However, the parcellation-based fMRI-radiomics analysis concerning spatial information of the whole brain characteristics might be suitable for applying to diseases with overall brain changes, rather than the gliomas with regional brain abnormality.

On the basis that rs-fMRI can provide a large number of imaging metrics describing pathophysiological processes of gliomas, here we intended to propose a novel omics analysis approach, named Regional Parameter of Resting-state fMRI-omics (RP-Rs-fMRIomics), by employing exhaustive regional parameters in a spectrum of specific frequency bands. Subsequently, we evaluated the diagnosis feasibilities of RP-Rs-fMRIomics in grading, molecular typing and prognosis on gliomas. We hypothesized that, in comparison with univariate analysis using single or finite imaging parameters [14,15,16], the RP-Rs-fMRIomics would ensure more precise diagnosis through comprehensive data analysis. On the other hand, in comparison with traditional radiomics with parcellation-based analysis [24,25], the RP-Rs-fMRIomics would be more readable with the explicit meaning of each feature as a certain rs-fMRI parameter at a certain frequency.

## 2. Material and Methods

### 2.1. Patients Enrollment

**Figure 1:**
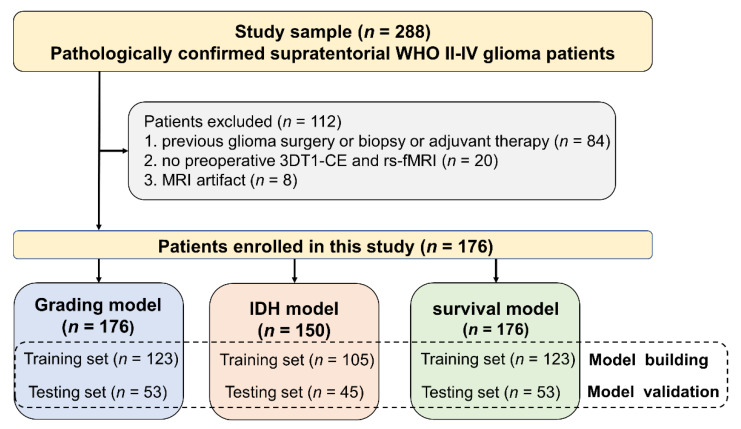
Flowchart of the inclusion and exclusion criteria for patients.

This retrospective study was approved by the Affiliated Jinling Hospital, Medical School of Nanjing University Medical Research Ethics Committee (protocol code 2019NZGKJ-083 and date of approval 5 March 2019). From December 2015 to February 2020, 288 consecutive patients pathologically confirmed for supratentorial WHO II-IV glioma were involved. Among them, 176 glioma patients who met the inclusion criteria were enrolled in this study (Figure 1).

### 2.2. Clinical Data

Glioma grades were diagnosed on histopathological examinations according to the 2007 or 2016 WHO classification criteria of central nervous system tumors [31,32]. High-grade gliomas (WHO III-IV) were found in 113 cases and low-grade gliomas (WHO II) were found in 63 cases. Isocitrate dehydrogenase (IDH) genetic diagnosis of gliomas was performed in 150 cases out of the 176 patients, with DNA sequencing (*n* = 112, Sanger sequencing or pyrosequencing) or immunohistochemistry (*n* = 38, antibody IDH1-R132H) on tumor samples. IDH wild/mutation types were found in 94/56 cases. The patients received partial (*n* = 86) or gross total (*n* = 90) resection followed by chemotherapy and radiation. Karnofsky Performance Status (KPS) scores (binary, score >70 or ≤70) [5,33], as a strong independent predictor of clinical outcome [34] determined by postoperative treatment, were retrieved from their electronic medical records. The demographic and clinical information are shown in Table 1.

### 2.3. MRI Data Acquisition

All patients underwent preoperative MR examinations on a 3.0-T scanner (Discovery MR750 System; GE Medical Systems, Milwaukee, WI, USA) with a 32-channel head coil. They were instructed to keep their eyes closed and stay awake.

Rs-MRI data were acquired using a gradient-echo planar imaging sequence (TR = 1000 ms, TE = 19 ms, FOV = 220 × 220 mm^2^, in-plane matrix = 64 × 64, flip angle = 75°, slice number = 20, slice thickness = 5 mm, interslice gap = 1.5 mm). Scan time lasted 6 min 45 s, and a total of 405 volumes were acquired.

High-resolution 3D T1WI images were obtained using a 3D-BRAVO sequence after contrast enhancement: TR = 8.2 ms, TE = 3.2 ms, matrix = 256 × 256, slice thickness = 1.0 mm and number of slices = 144. Moreover, routine MR images including pre- and post-enhanced T1WI, T2WI, T2-FLAIR weighted imaging were acquired for radiological diagnosis. About 0.1 mmol/kg of gadolinium chelate contrast was injected for contrast-enhanced imaging.

### 2.4. Imaging Processing

#### 2.4.1. Data Preprocessing

Rs-fMRI data were preprocessed using SPM 12 (http://www.fil.ion.ucl.ac.uk/spm (access on 8 April 2021))-based toolkit of DPARSF toolbox (DPARSF_V2.3; www.restfmri.net (access on 8 April 2021)) [35], including: (1) discarding the first 5 volumes; (2) slice timing correction; (3) head motion correction; (4) registering to individual 3DT1-CE images; (5) smoothing with a 6 mm FWHM isotropic Gaussian kernel (except for ReHo calculation; for the calculation of Hurst and TSA, including smooth and non-smooth); (6) removing covariates of head motion, white matter signal and cerebrospinal fluid; (7) considering the physiological information contained in potential specificity in different frequency bands, in addition to the frequency range (0.01–0.1 Hz) of BOLD fluctuation, we also divided them into 9 narrow band bins, i.e., each narrow frequency band covered 0.01 Hz. Based on these 10 frequency bands, we reckoned that the measures on the pinpoint frequency fluctuation would increase as much of the information about the BOLD fluctuations and find the optimal frequency sub-band of BOLD effects in gliomas

To comprehensively delineate the features of the BOLD signals within the tumor, we calculated the regional rs-fMRI parameters as much as available currently, including four main parameters: ReHo [8,9], ALFF [7], HE [10,36,37,38] and TSA [39]. Moreover, fraction of ALFF (fALFF) [7,40] as a commonly used variant of ALFF was also calculated. The definition of each parameter is presented in Supplementary Material S1. ReHo and ALFF analyses were performed using rs-fMRI Data Analysis Toolkit (http://resting-fmri.sourceforge.net (accessed on 8 April 2021)), while HE and TSA were calculated using an in-house toolkit in line with methods in Lei et al. [41] and Lv et al. [42]. Moreover, considering the controversy about the physiological meaning of the global signal, we further calculated the above parameters after global regression.

#### 2.4.2. Tumor Segmentation and Feature Extraction

**Figure 2:**
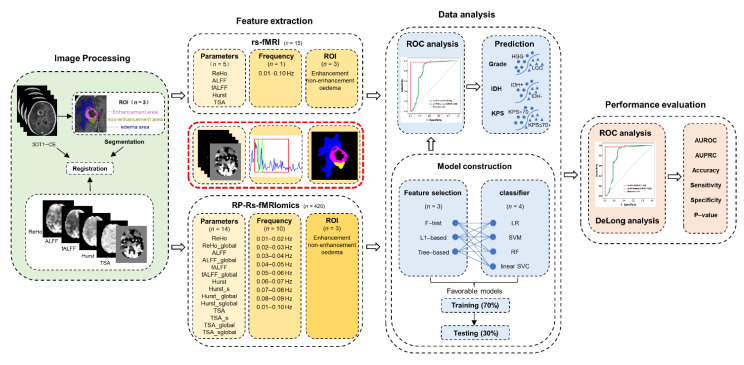
The whole workflow of this study. RP-Rs-fMRIomics models were constructed for predicting glioma grade, isocitrate dehydrogenase status and Karnofsky Performance Status scores, based on the features extracted through measuring 14 regional parameters of rs-fMRI in 10 specific narrow frequency bins and 3 parts of gliomas. Then, three radiomics feature selections and four classifiers were implemented. The diagnostic performances of RP-Rs-fMRIomics models were compared with conventional single-parameter fMRI analysis using Delong’s test.

Blinded to all the information of patients, two experienced neuroradiologists manually segmented the tumors into three subregions (enhancement, non-enhancement and peritumor edema areas) slice by slice on 3DT1-CE images using MRIcron software (http://www.nitric.org/projects/mricron (access on 5 February 2021)) (Figure 2). The segmentation of these three subregions refers to the Multimodal Brain Tumor Image Segmentation Benchmark [43]. Then, the regions of interest (ROIs) were co-registered with functional data and normalized to MNI coordinates.

Finally, we adopted 420 features at three levels: 14 parameters (ALFF, fALFF, ReHo, HE, and TSA with and without global regression, and HE, TSA with and without smooth), measured in 10 specific frequency bands, and the values extracted within 3 regions.

### 2.5. Statistical Analysis

#### 2.5.1. Conventional rs-fMRI Analysis

We calculated the regional parameters in the conventional strategy within frequency at 0.01~0.1Hz in order to perform prediction analysis of each rs-fMRI parameter on gliomas. Receiver operating characteristic (ROC) analysis was performed to measure the diagnostic performance of these indexes based on ROIs for tumor grading, IDH status and prognosis in gliomas. According to the AUC, the best index was chosen for each task.

#### 2.5.2. Feature Selection, RP-Rs-fMRIomics Model Construction and Validation

With 420 features extracted from rs-fMRI, we constructed RP-Rs-fMRIomics models for (1) glioma grades, (2) IDH genotype and (3) prognosis. Our dataset was randomly split into a training set and a testing set (ratio 7:3). A few multiple machine-learning classifiers, including Logistic Regression (LR), Support Vector Machine (SVM), Random Forest (RF), and Linear support vector classification (Linear SVC) were used. Firstly, we used Spearman correlation analysis to alleviate the redundancy between the features. When the linear correlation coefficient between any two independent variables on the training set was greater than 0.9, a feature would be removed. The features which had a high linear correlation coefficient with the dependent variables were preferentially retained. Then, three feature selection methods of F-test, L1-based feature selection with linear models and Tree-based feature selection were utilized for model building.

Fivefold cross-validation and a grid search with F1 score as the optimization goal were carried out on the training set to tune the model hyperparameters, and the performances were tested on the testing set. Considering the area under the receiver operating characteristic (AUROC) [44] and precision−recall curves (AUPRC) [45] could display the accuracy, precision and recall of the model in a more comprehensive way with varying thresholds, an optimal model with the top AUROC and the highest AUPRC was selected for each prediction task. We also performed comparisons of diagnosis performance between each optimal model of conventional rs-fMRI and RP-Rs-fMRIomics using the Delong’s test [46].

## 3. Results

### 3.1. Patient Characteristics

There was no significant difference in age, histopathologic grade, IDH status and extent of resection between training and validation sets in the three models (grading, IDH and survival model). The gender of the IDH model and the KPS of the grading and survival models were not statistically different in the training and validation sets (all *p* > 0.05).

However, the validation set showed a higher proportion of male patients in the grading and survival model (*p* = 0.019, 0.008) and a higher proportion of patients with KPS > 70 in the IDH model (*p* = 0.001) (Table 1).

### 3.2. Performance of Conventional rs-fMRI Analysis

**Figure 3:**
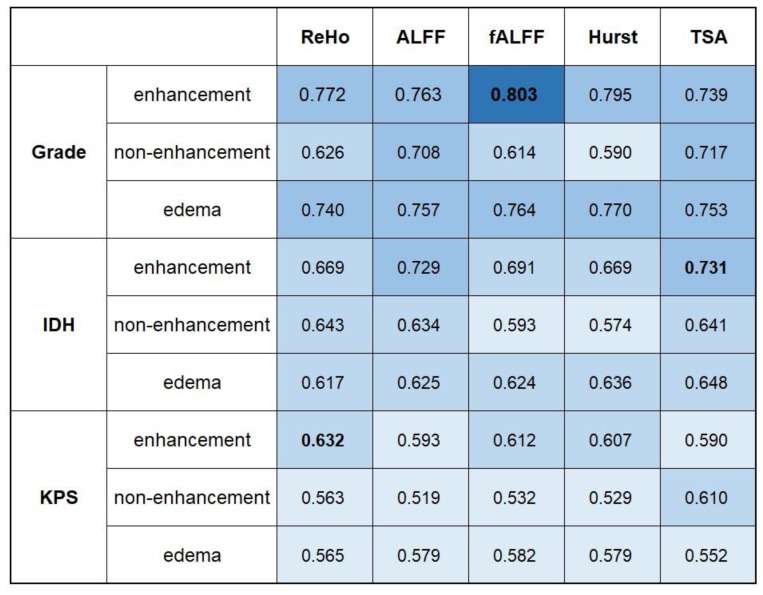
Heatmap depicting the differentiating power of conventional rs-fMRI parameters (rows) with the AUROC based on the three ROIs (columns) in the grading model, IDH model and survival model.

**Figure 4:**
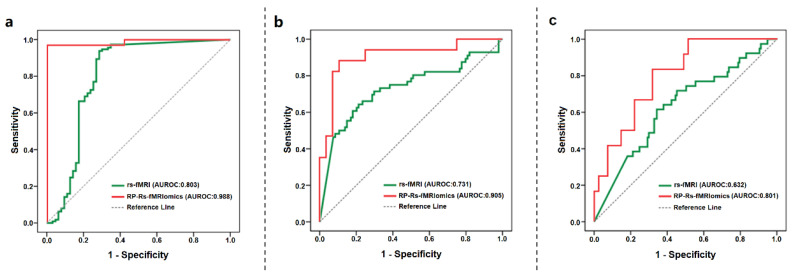
Receiver operating characteristic (ROC) curves for three prediction models in rs-fMRI and in the validation cohort of RP-Rs-fMRIomics: (**a**) grading model; (**b**) IDH grade model; (**c**) survival model.

The AUROCs of five conventional indexes based on three ROIs were calculated to quantify the predictive performance of these indexes (Figure 3). According to the AUROC, fALFF, TSA and ReHo based on enhancement ROI achieved slightly higher AUCs of 0.803, 0.731 and 0.632 in tumor grading, IDH status and progression models, respectively. However, there was no statistical significance of the improvement in AUCs above compared with other indexes based on different ROIs (*p* > 0.0036 for all). The specific *p* value was shown in Supplementary Materials Table S2. This might be related to our limited amount of data. Therefore, we still chose fALFF, TSA and ReHo based on enhancement ROI as the best models for predicting tumor grading, IDH status and progression, and the ROC curves are presented in Figure 4.

### 3.3. Performance of RP-Rs-fMRIomics Models

The optimal model for each task was chosen on the training set and validated on the testing set. The RP-Rs-fMRIomics with the optimal hyperparameter configuration showed outstanding performance to predict grading (AUROC 0.988, AUPRC 0.971), IDH (AUROC 0.905, AUPRC 0.824), and survival (AUROC 0.801, AUPRC 0.667) (Table 2). The grading and IDH models combining RF and F test and the survival model combining LR and F test achieved the best classification performances. The hyperparameters search ranges and optimal sets in each model are presented in Supplementary Material S3. The ROC curves of the three models are presented in Figure 4.

### 3.4. Key Imaging Features in RP-Rs-fMRIomics Models

**Figure 5:**
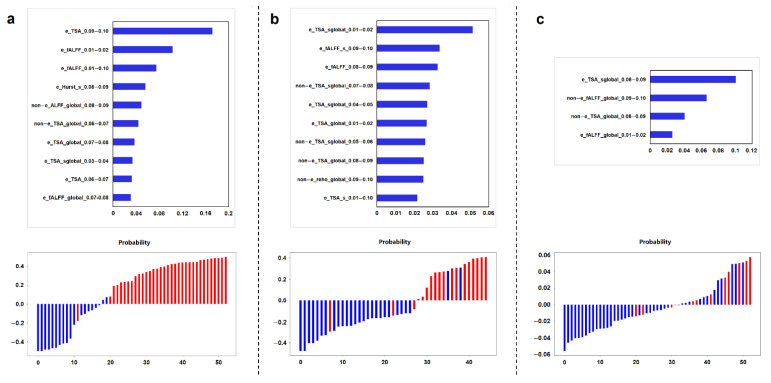
The first row shows the top 10, 10 and 4 importance ranking of RP-Rs-fMRIomics features in the (**a**) grading model, (**b**) IDH model and (**c**) survival model, respectively. The second row shows the bar plots of these three models in the validation cohort. The red bars with the predictive value >0 and the purple bars with the predictive value <0 indicate the successful classification of the corresponding prediction model, and vice versa.

In the grading model, IDH model and survival models, 59 features, 73 features and 4 features were selected, respectively. A detailed feature list of each RP-Rs-fMRIomics model can be found in Supplementary Material Table S4. Figure 5 shows the top 10 importance ranking of RP-Rs-fMRIomics features in both the grading model and IDH model, and 4 importance ranking of RP-Rs-fMRIomics features in the survival model. In terms of parameters, what stood out in the result was TSA-based RP-Rs-fMRIomics features, and they had a large proportion in each model (61.02%, 67.12%, 50.00%). In terms of space, the features of the enhancement area accounted for a large proportion in each model (62.71%, 53.42%, 50.00%). In terms of frequency, RP-Rs-fMRIomics features in the grading model and IDH model were relatively balanced in ten specific frequency bands. However, the high-frequency features distributed between 0.08–0.10 Hz were useful for predicting the prognosis of glioma patients.

### 3.5. Comparisons of Prediction Performance between Conventional rs-fMRI and RP-Rs-fMRIomics Model

The AUROC of each optimal RP-Rs-fMRIomics model out-performed the corresponding best conventional rs-fMRI index for three prediction tasks (*p* < 0.0001 in grading model and IDH model, *p* = 0.0044 in survival model). For tumor grading, two of the first three features of RP-Rs-fMRIomics model were derived from the fALFF parameter, which also performed superiorly in conventional rs-fMRI. In terms of space, whether in the conventional rs-fMRI or RP-Rs-fMRIomics model, the features of the tumor enhancement area were very important for each clinical task.

## 4. Discussion

In this work, based on a large number of regional parameters of rs-fMRI comprehensively and meticulously extracted in specific parts of gliomas, we innovatively introduced omics analysis and proposed a novel concept of RP-Rs-fMRIomics analysis. With a pilot study by applying the method to prediction diagnosis of brain gliomas, we found that the method presented superior performance in tumor grade, molecular type and prognosis of brain gliomas, with significant improvement relative to the conventional rs-fMRI methods. Featuring high interpretability, this RP-Rs-fMRIomics contributed a new method for brain tumor research and also expanded the clinical application of rs-fMRI.

By proposing a novel technique of RP-Rs-fMRIomics, we extract comprehensive rs-fMRI features for clinical prediction. Many previous studies [5,14,15,47] have adopted only limited parameters in specific frequency bands, according to the researchers’ best prior knowledge, inevitably neglecting other potentially useful information. On the other hand, our RP-Rs-fMRIomics has the advantage of full coverage of rs-fMRI indexes, including parameters, frequency and space information, and can reflect rich physiological information. Moreover, in previous studies [48,49,50], the common radiomics analysis strategy was making various clinical predictions based on tumor attributes and engineering complex image descriptors inside or nearby the tumors. It is difficult to fully understand the biological meaning of the numerical data derived from the analysis, such as texture features [51] and wavelet transform features [52]. Compared with radiomics, the features of RP-Rs-fMRIomics are more interpretable, which can well define the specific parameter in a specific frequency band and lesion area. Recently, functional connectomics, based on the combination of radiomics and functional connectivity, has been used to predict individualized overall survival time of glioma patients [27]. However, to some extent, the network metrics at the whole brain level lack a certain logic in reflecting the attributes of the tumor itself because of spatial heterogeneity in gliomas. In contrast, our RP-Rs-fMRIomics would be more logical for using regional parameters within the tumor to construct models.

Of note, as a prediction study, our RP-Rs-fMRIomics has achieved excellent performance in predicting tumor grade, molecular type and prognosis in gliomas, compared with conventional rs-fMRI. Several rs-fMRI studies [14,15,16] showed that quantification of spontaneous fluctuations—such as parameters power spectra, signal intensity correlation, ALFF, fALFF and TSA—had the potential for distinguishing between high- and low-grade gliomas (AUROC:0.67–0.89). These results were similar to our conventional rs-fMRI, but the AUROCs were significantly lower than those of our RP-Rs-fMRIomics. Additionally, Englander et al. [47] observed greater vascular dysfunction outside the identifiable margins in IDH wide-type tumors than in IDH mutant tumors, and showed that rs-fMRI was highly predictive of the IDH mutation. Petridis et al. [18] used rs-fMRI to measure asynchrony in vascular dynamics measured to quantify the tumor burden and infiltration degree in IDH-mutated gliomas. In accordance with previously reported research, patients with high postoperative KPS (KPS > 70) had longer survival times. Daniel et al. [5] and Liu et al. [27] have highlighted that the rs-fMRI and the strength of functional connectivity have prognostic value and can predict the overall survival time of gliomas. Relative to the previous studies [53,54,55,56,57,58,59], our RP-Rs-fMRIomics models for predicting tumor grading and IDH status presented comparable or even better performances to the radiomics models. Hence, our study indicated that RP-Rs-fMRIomics could be a promising application in various clinical predictions of gliomas.

With significantly increased prediction accuracy of tumor grading, IDH status and KPS, we can further analyze which RP-Rs-fMRIomics features could contribute to these predictions and why. Our results showed that TSA-based RP-Rs-fMRIomics features and enhancement region-based features were more prominent in various prediction models. As previous studies reported, BOLD signals contain not only information about local blood flow but also oxygen consumption [60], which can be used to evaluate the damaged hemodynamic status or severity [61]. TSA has recently been used to assess pathophysiological events related to hemodynamics [42,61,62] and provides a high spatial correspondence with the hypoperfusion area defined by DSC-PWI on the individual level. Gupta et al. [16] tried to use TSA to characterize tumor vascularity in gliomas by observing the aberrant dynamics of tumor vascular and corresponding blood perfusion. They found that TSA was significantly different between high-grade and low-grade, with advanced TSA in high-grade gliomas. Because the increase in tumor vascularization and the decrease in microvascular blood flow resistance may advance hemodynamics, TSA plays an important role in tumor grading, IDH status and prognosis prediction. Many studies [63,64,65] have revealed the microenvironmental heterogeneity in gliomas, such as histologic heterogeneity composed of tumor cells and different stromal cells, and cell heterogeneity with extensive genetic and epigenetic variations. Recent studies [56,66,67,68] have highlighted the value of multiregional image analysis. Li et al. [57] found that, for predicting glioblastoma IDH1 status, the multiregional model built with all region features outperformed the single region models. However, in the single region model, the model constructed by enhancement area achieved the highest AUROC in the primary cohort. The result of this part is consistent with that of our research. The contrast enhancement indicates an interruption and leakage of the blood−brain barrier, which is mainly found in the area of a highly vascular tumor [69,70]. Compared with other regions, the enhancement area may better reflect the pathophysiological mechanism of the tumor and is conducive to clinical evaluation.

Several limitations should be noted. First, our study only employed a single-center dataset. Independent data from multiple centers were warranted for external validation. Second, the ten specific frequency divisions for BOLD signals were arbitrary. Third, we only employed the regional parameters of rs-fMRI that were commonly used and were within the reach of our ability. Finally, this was a pilot application study, and further studies are needed to better understand this disease.

## 5. Conclusions

Based on exhaustive regional parameters of rs-fMRI and by adopting an omics analysis tactic, we proposed an RP-Rs-fMRIomics analysis. With an entire investigation of the data and the high interpretability of the results, the RP-Rs-fMRIomics outperformed traditional rs-fMRI in the prediction of tumor grade, molecular type and prognosis of gliomas. The RP-Rs-fMRIomics expanded the clinical application of rs-fMRI and could be used as a novel strategy for the diagnosis of gliomas.

## Figures and Tables

**Table 1:** Demographic and clinical data of glioma patients.

Variables	Grading Model				IDH Model				Survival Model			
	All Patients (*n* = 176)	Training Set (*n* = 123)	Testing Set (*n* = 53)	*p* Value	All Patients (*n* = 150)	Training Set (*n* = 105)	Testing Set (*n* = 45)	*p* Value	All Patients (*n* = 176)	Training Set (*n* = 123)	Testing Set (*n* = 53)	*p* Value
Age (±SD), years	51.11 ± 13.74	51.67 ± 12.24	49.83 ± 16.77	0.474	50.65 ± 13.37	52.31 ± 14.53	50.44 ± 12.43	0.503	51.11 ± 13.74	50.97 ± 13.54	51.45 ± 14.32	0.830
Gender				0.019 *				0.390				0.008 *
male	96	60	36		82	55	27		96	59	37	
female	80	63	17		68	50	18		80	64	16	
WHO grade				0.992				0.682				0.308
II	63	44	19		53	36	17		63	47	16	
III-IV	113	79	34		97	69	28		113	76	37	
IDH status				0.242				0.941				0.528
Mutant	56	38	18		56	39	17		56	42	14	
Wild type	94	72	22		94	66	28		94	66	28	
Extent of resection				0.070				0.669				0.107
gross-total	90	57	33		76	52	24		90	58	32	
partial	86	66	20		74	53	21		86	65	21	
KPS				0.198				0.001 *				0.619
>70	39	24	15		32	15	17		39	26	13	
≤70	137	99	38		118	90	28		137	97	40	

Abbreviations: WHO = World Health Organization, IDH = isocitrate dehydrogenase, KPS = Karnofsky Performance Status. * represents the number of *p*-values < 0.05.

**Table 2:** Prediction performance of grading, IDH and survival models.

Optimal Model		Grading Model	IDH Model	Survival Model
Classifier		Random Forest	Random Forest	Logistic Regression
Feature Selection		F Test	F Test	F Test
Training set	AUROC	0.999	1.000	0.706
	ACC	0.984	0.991	0.642
	AUPRC	0.987	1.000	0.667
	SEN	0.987	1.000	0.667
	SPE	0.977	0.985	0.635
	F1 score	0.987	0.987	0.450
Testing set	AUROC	0.988	0.905	0.801
	ACC	0.943	0.867	0.698
	AUPRC	0.971	0.824	0.667
	SEN	0.971	0.824	0.667
	SPE	0.895	0.893	0.707
	F1 score	0.957	0.824	0.500

Abbreviations: AUROC = area under the receiver operating characteristics; ACC = accuracy; AUPRC = area under the precision−recall curve; SEN = sensitivity; SPE = specificity.

## Data Availability

The datasets used in the current study are available from the corresponding author on reasonable request.

## Supplementary Materials

The following supporting information can be downloaded at: https://www.mdpi.com/article/10.3390/cancers14122818/s1, Supplementary Material S1: The definition of regional rs-fMRI parameters, Table S1: *p* values of DeLong analysis among the AUROCs of five conventional indexes based on three ROIs, Supplementary Material S2: Hyperparameters for model optimization, Table S2: Detailed imaging features in RP-Rs-fMRIomics models.
