# Supplementary material for: RP-Rs-fMRIomics as a Novel Imaging Analysis Strategy to Empower Diagnosis of Brain Gliomas
Source: Cancers (Basel). 2022 Jun 7;14(12):2818. doi: 10.3390/cancers14122818 (PMC9220978; doi:10.3390/cancers14122818)
Supplement: Supplementary file 1 [file cancers-14-02818-s001.zip › supplementary material S1.pdf]

**The Definition of Regional rs-fMRI Parameters**

(1) Regional homogeneity (ReHo) is defined as the Kendall's W between the time series of a given voxel and its nearest voxel time series, which represents the local synchronicity of neural activations in the same functional cluster.

(2) Amplitude of low-frequency fluctuation (ALFF) is defined as the mean value of the Fourier transform amplitude of an fMRI time series in a specific frequency range. Moreover, fraction of ALFF (fALFF) as a commonly used variant of ALFF is the fraction of ALFF in a given frequency band to the ALFF over the entire frequency range of the fMRI signal.

(3) Hurst exponent (HE) measures scale-free properties of intrinsic brain activity and reflects temporal complexity.

(4) Time-shift-analysis (TSA) was defined as the temporal shift required to the maximal correlation with reference time course (i.e., the global mean of the brain).
