# Supplementary material for: RP-Rs-fMRIomics as a Novel Imaging Analysis Strategy to Empower Diagnosis of Brain Gliomas
Source: Cancers (Basel). 2022 Jun 7;14(12):2818. doi: 10.3390/cancers14122818 (PMC9220978; doi:10.3390/cancers14122818)
Supplement: Supplementary file 1 [file cancers-14-02818-s001.zip › supplementary material S2.pdf]

The Hyperparameters Tuned Included:

Selector:

(1) F-test: percentage: 1, 5, 10, 30, 50, 100
(2) L1-based: C: 0.1, 1, 10
(3) Tree-based:

Classifier:

1. Logistic Regression:
    a. penalty: ["l1", "l2"]
    b. C: C_dist
    c. "fit_intercept": [True, False]
    d. "class_weight": ["balanced", None]
2. SVM
    a. kernel: ["linear", "rbf", "poly", "sigmoid"]
    b. C: C_dist
    c. class_weight: ["balanced", None]
3. LinearSVC
    a. penalty: ["l2"]
    b. C: C_dist
    c. class_weight: ["balanced", None]
    d. dual: [True, False]
4. RandomForest
    a. criterion: ["gini", "entropy"]
    b. n_estimators: st.randint(low=10, high=100)
    c. min_samples_split: st.randint(low=3, high=10)
    d. max_features: ['auto', 'sqrt', 'log2', None]
    e. bootstrap: [True, False]
    f. class_weight: ["balanced", None]

1. Optimal sets in grading model

Selector: f_classif, percentage,50
Classifier: RandomForest
'classifier__criterion': 'gini'
'classifier__n_estimators': 60
'classifier__min_samples_split': 6
'classifier__max_features': None
'classifier__bootstrap': False,
'classifier__class_weight': 'balanced'

2. Optimal Sets in IDH Model

Selector: f_classif, percentage,50

Classifier: RandomForest
'classifier__criterion': 'entropy'
'classifier__n_estimators': 22
'classifier__min_samples_split': 4
'classifier__max_features': 'sqrt'
'classifier__bootstrap': True
'classifier__class_weight': None

3. Optimal Sets in Survival Model

Selector: f_classif, percentage,50
Classifier: LogisticRegression
'classifier__penalty': 'l1'
'classifier__C': 0.14079999999999998
'classifier__class_weight': None
'classifier__fit_intercept': False
